# Outpatient treatment following alcohol screening at health checkups and change in drinking patterns among excessive drinkers with lifestyle-related diseases

**DOI:** 10.1016/j.pmedr.2021.101549

**Published:** 2021-09-08

**Authors:** Ayumi Takano, Hayato Yamana, Sachiko Ono, Hiroki Matsui, Hideo Yasunaga

**Affiliations:** a Department of Mental Health and Psychiatric Nursing, Tokyo Medical and Dental University, 1-5-45 Yushima, Bunkyo-Ku, Tokyo, Japan; b Department of Health Services Research, The University of Tokyo, 7-3-1 Hongo, Bunkyo-Ku, Tokyo, Japan; c Department of Eat-loss Medicine, The University of Tokyo, 7-3-1 Hongo, Bunkyo-Ku, Tokyo, Japan; d Department of Clinical Epidemiology and Health Economics, The University of Tokyo, 7-3-1 Hongo, Bunkyo-Ku, Tokyo, Japan

**Keywords:** Hazardous and harmful alcohol use, Screening and brief intervention, Health checkup, Health insurance database, Propensity score matching

## Abstract

Screening and brief interventions have been implemented in primary care settings to reduce excessive drinking. However, the effectiveness of screening and brief interventions within a health checkup setting is unclear. We assessed the effectiveness of general treatment for lifestyle-related diseases following screening for alcohol consumption at health checkups, using data on medical claims and health checkups. Participants were people with excessive drinking who met the threshold of recommendation to receive treatment for lifestyle-related diseases. We analyzed risky drinkers (drinking every day, 40–60 g/day for male and 20–60 g/day for female) and heavy drinkers (drinking every day, >60 g/day) separately. We performed one-to-one propensity score matching between people who received general outpatient treatment for lifestyle-related diseases and those who did not. Outcomes were drinking patterns (frequency and amount) and liver function at the next year's health checkup. Middle-aged males accounted for about 94% of the 23,347 participants. Eleven percent of the participants received treatment after the health checkup. After propensity score matching, among 1990 pairs of risky drinkers, those with treatment were significantly more likely to reduce their frequency of drinking (11.7% vs. 8.7%, p = 0.002) and showed lower transaminase values than those without treatment. In 575 pairs of heavy drinkers, there was no significant change in drinking patterns or liver function. Treatment in primary care after screening at health checkups was associated with the change in drinking behavior and improvement in liver condition among risky drinkers. More intensive intervention may be needed to reduce drinking in heavy drinkers.

## Highlights

• General outpatient care following alcohol screening at health checkups was evaluated.
• Database of claims data and health checkups was used for propensity score matching.
• Outpatient care was associated with reduced drinking frequency in risky drinkers.
• Outpatient care was not associated with improved drinking behavior in heavy drinkers.

## 1 Introduction

Excessive alcohol use is a significant cause of mortality, morbidity and social problems. Alcohol consumption causes digestive diseases, cardiovascular diseases, and cancers (World Health Organization, 2018). Health risks associated with alcohol consumption rise along with an increase in the amount of daily drinking (Day and Rudd, 2019, Degenhardt et al., 2018). Although alcohol dependence is a severe alcohol-related problem, most alcohol-related problems occur in a large number of drinkers who are not alcohol-dependent (Anderson, 1991). Therefore, a population-based approach for drinkers has a significant impact on reduction in overall alcohol-related harm (Knox et al., 2019, World Health Organization, 2001).

Screening and brief interventions (SBI) in primary care settings have been implemented as a strategy to reduce hazardous or harmful drinking (World Health Organization, 2001, World Health Organization, 2018, Kaner et al., 2009, Beyer et al., 2019, Knox et al., 2019). A systematic review of trials of SBI provided at general healthcare facilities, emergency departments, and trauma centers revealed that people who received SBI consumed less alcohol and reduced their frequency of drinking than participants with minimal or no intervention after one year (Kaner et al., 2018). The systematic review also revealed that extended intervention, which was comprised of either more than five sessions or more than 60 min in total and based on motivational interviewing or cognitive behavioral therapy, had a limited impact compared with standard SBI (Kaner et al., 2018).

In Japan, employers are obliged to provide annual health checkups for their employees based on the *Industrial Safety and Health Act*. Employees have this mandatory health checkup at least once a year, and the participation rate among employees is approximately 80% (Ministry of Health and Labour and Welfare, 2008). One of the objectives of this health checkup is early detection and intervention for lifestyle-related diseases among workers. The standard health checkup includes height and weight measurements, blood pressure measurement, vision and hearing tests, blood tests including liver function, urinary tests, and chest X-ray (Ministry of Health, Labour and Welfare, 2018). Lifestyle and habits, including drinking patterns, are also assessed using standardized self-reported questionnaires (Ministry of Health, Labour and Welfare, 2018). If a person is diagnosed as being at risk of lifestyle-related diseases, recommendations are made to receive treatment or health guidance at outpatient services. Although this system of health checkups and recommendations may allow early intervention for hazardous or harmful drinkers who are also at risk for lifestyle-related diseases, its effectiveness has not been evaluated. Additionally, most studies assessed the effectiveness of SBI using self-reported alcohol drinking levels (Kaner et al., 2009; 2018; Schmidt et al., 2016). Laboratory findings should also be used to evaluate the effect of SBI.

The aim of this study was to assess the effectiveness of general outpatient treatment following alcohol consumption screening at health checkups. The population of interest was risky or heavy drinkers who also met the threshold to be given a recommendation to seek outpatient treatment for lifestyle-related diseases. We utilized a large-scale database of health checkups and medical claims data and used self-reported alcohol consumption and laboratory findings as outcomes.

## 2 Methods

### 2.1 Data source

A retrospective cohort study was performed using the JMDC Claims Database, a database of health checkups and medical claims constructed by JMDC Inc. (Tokyo, Japan). JMDC Inc. has collected claims information from occupation-based health insurance agencies for corporate employees and their dependents since 2005 (Kimura et al., 2010). The JMDC Claims Database includes anonymous data of inpatient, outpatient, and pharmacy claims and health checkups from about 7,300,000 individuals as of April 2020 (JMDC Inc., 2020). The database includes information about patient characteristics, diagnoses, drug prescriptions, medical procedures, characteristics of medical facilities, and reimbursement fees. The diagnoses are based on the International Classification of Diseases, 10th revision (ICD-10) diagnostic codes. The health checkup data consist of results for anthropometric measurements, blood tests, medical interviews, and lifestyle questionnaires, including questions about alcohol drinking patterns (frequency [none, rarely, sometimes, and every day] and consumption per day) and motivation to change lifestyle. Alcohol consumption per day is assessed in the questionnaire by a glass of sake (approximately 20 g of pure ethanol) as a unit and as a reference for converting from other types of alcohol. This categorization is based on the results of a previous cohort study (Tsugane et al., 1999) and is also utilized in the “*Health Japan 21*″ policy (Ministry of Health, Labour and Welfare, 2012). We converted this into <20 g, 20–40 g, 40–60 g, >60 g of ethanol per day. We used data from April 1st, 2015, through March 31st, 2018.

The requirement for informed consent was waived because of the anonymous nature of the data. The study was approved by the Institutional Review Board of The University of Tokyo (No. 10862).

### 2.2 Participant selection

We first identified individuals who received a health checkup in 2016. If an individual received two or more health checkups in a year, we selected data from the first health checkup. Participants were selected if they fit the following inclusion criteria: 1) insured employees, 2) continuous enrollment in health insurance from April 2015 to March 2018, 3) alcohol consumption based on the 2016 health checkup, and 4) meeting the threshold of recommendation to receive treatment for lifestyle-related diseases (high blood pressure, diabetes, liver diseases, dyslipidemia, or hyperuricemia) during the 2016 health checkup. The thresholds were based on health checkup guidelines (Ministry of Health, Labour and Welfare, 2018): systolic blood pressure ≥ 140 mmHg, diastolic blood pressure ≥ 90 mmHg, hemoglobin A1c (HbA1c) ≥ 6.5%, fasting blood glucose ≥ 126 mg/dL, casual blood glucose ≥ 126 mg/dL, glutamate oxaloacetate transaminase (GOT) ≥ 51 U/L, glutamate pyruvate transaminase (GPT) ≥ 51 U/L, gamma-glutamyl transpeptidase (γ-GT) ≥ 101 U/L, triglycerides ≥ 300 mg/dL, high-density lipoprotein (HDL) ≤ 34 mg/dL, low-density lipoprotein (LDL) ≥ 140 mg/dL, or uric acid ≥ 8 mg/dL. We excluded those who had received treatment for lifestyle-related diseases at any institution during one year preceding the 2016 health checkup, those who had received treatment for alcohol-related diseases at psychiatry during one year preceding the 2016 health checkup, those without data from a 2017 health checkup, and those with missing data on alcohol consumption or frequency in the 2017 health checkup.

We divided the people into three groups: low-risk drinkers (not drinking every day, or drinking < 40 g/day for male and < 20 g/day for female), risky drinkers (drinking every day at 40–60 g/day for male and 20–60 g/day for female), or heavy drinkers (drinking every day at > 60 g/day) (Ministry of Health, Labour and Welfare, 2012). We included risky drinkers or heavy drinkers for the analysis separately.

### 2.3 Measures

The primary outcome was alcohol drinking patterns (frequency and consumption per day) assessed at the 2017 health checkup. The frequency of alcohol drinking and the alcohol consumption per day was assessed using three categories (no or rarely, sometimes, or every day) and four classes (<20 g, 20–40 g, 40–60 g, or ≥60 g), respectively. We identified whether participants improved in their drinking patterns or not. Improvement in frequency was defined by the frequency as: sometimes, rarely, or none in 2017. Participants were considered to have improved alcohol consumption when the category of consumption in 2017 was lower than that in 2016. For example, in risky male drinkers, those who reduced the amount of drinking to less than 40 g per day in 2017 were considered to have improved. In heavy drinkers, those who reduced the amount to less than 60 g per day were considered to have improved in both males and females. The secondary outcome was laboratory results associated with liver conditions in 2017 (GOT, GPT, and γ-GT). We also defined liver dysfunction in 2017 (binary variable) as at least one of GOT, GPT, or γ-GT meeting the threshold of recommendation for treatment.

The exposure variable was whether participants received outpatient treatment for lifestyle-related diseases between the 2016 and 2017 health checkups or not (treatment or no treatment). We identified outpatient treatment for lifestyle-related diseases or outpatient nutrition guidance using claim records of a specific reimbursement for the continuous outpatient management of lifestyle-related diseases conducted by primary care physicians at clinics or small hospitals (<200 beds). To receive this reimbursement, primary care physicians must provide patients with comprehensive treatment and health guidance, such as medication and advice for exercise or nutrition.

We assessed the following variables as potential predictors of receiving outpatient treatment. Participant characteristics included sex and age. Health conditions were evaluated using blood pressure, body mass index (BMI), abdominal circumference, results of blood test (HbA1c, fasting blood glucose, casual blood glucose, GOT, GPT, γ-GT, triglycerides, HDL, LDL, and uric acid), and conditions of recommendation to receive treatment (systolic blood pressure, diastolic blood pressure, HbA1c, fasting blood glucose, casual blood glucose, GOT, GPT, γ-GT, triglycerides, HDL, LDL, and uric acid). The motivation for lifestyle change was also assessed using two questions: intention to change lifestyles such as exercise and diet (no intention, intention to change within 6 months, intention to change within one month, changed and maintained for <6 months, changed and maintained for ≥6 months, or missing), and intention to receive health guidance about the lifestyle improvement (yes, no, or missing).

### 2.4 Statistical analysis

We analyzed risky drinkers and heavy drinkers separately. Propensity scores were utilized to balance the backgrounds between the groups (treatment or no treatment). In order to estimate the propensity score, logistic regression models were constructed with the treatment condition as the dependent variable and the following factors as independent variables: participant characteristics, motivation for lifestyle change, blood pressure, BMI, abdominal circumference, results of blood test (triglycerides, HDL, LDL, GOT, GPT, γGT), and conditions of recommendation to receive treatment. People with missing data for the calculation of propensity score were excluded from the analysis. Propensity score matching was performed using 1:1 nearest-neighbor matching without replacement with a caliper distance of 0.2 for the standard deviation of the propensity score. We confirmed standardized differences between the groups before and after the propensity score matching. A standardized difference of less than 0.1 was considered indicative of balance. Chi-squared test and Student's *t*-test were used to compare categorical and continuous outcomes, respectively.

The threshold for significance was p < 0.05. Stata version 16 (StataCorp, College Station, TX, USA) was used for all statistical analyses.

## 3 Results

### 3.1 Participant characteristics

**Fig. 1:**
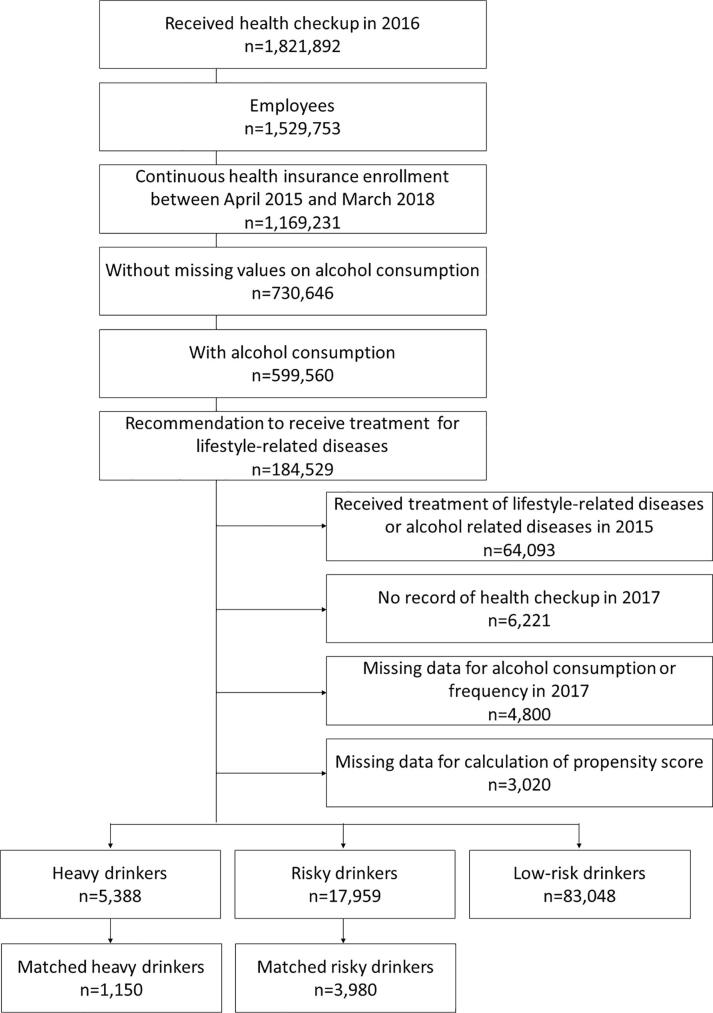
Participant selection process.

Fig. 1 shows participant selection. We identified 1,821,892 individuals (1,191,634 males, 630,258 females) who received health checkup in 2016. Among 599,560 participants (514,030 males, 85,530 females) with alcohol consumption, 184,529 (173,727 males, 10,802 females) met the threshold for recommendation to receive treatment for lifestyle-related diseases. We excluded those who had received treatment for lifestyle-related or alcohol-related diseases during the one year preceding the 2016 health checkup (n = 64,093) and those without data from the 2017 health checkup (n = 6221). Moreover, we excluded people with any missing data of variables for alcohol consumption or frequency in 2017 (n = 4800) and variables required for the calculation of the propensity score (n = 3020). We included 23,347 individuals for analysis (17,959 risky drinkers and 5388 heavy drinkers) after excluding low-risk drinkers (n = 83,048).

Table 1 shows the participant characteristics in both risky drinkers and heavy drinkers before propensity score matching. Among the 17,959 risky drinkers, 1991 individuals (11.1%) received treatment for lifestyle-related diseases between the 2016 and 2017 health checkups. Among the 5388 heavy drinkers, 579 individuals (10.7%) received treatment. In both populations, a greater proportion of the treatment group met the threshold of high blood pressure and high HbA1c compared with the no treatment group. Detailed results of the blood test before propensity score matching are shown in Supplementary Table 1, and the outcomes before propensity score matching are shown in Supplementary Table 2.

**Table 1:** Demographic characteristics and health conditions at the first health checkup before propensity score matching.

		Risky drinker (n = 17,959)					Heavy drinker (n = 5,388)				
		Treatment		No treatment			Treatment		No treatment		
		(n = 1,991)		(n = 15,968)		Standardized	(n = 579)		(n = 4,809)		Standardized
		n/mean	%/SD	n/mean	%/SD	difference	n/mean	%/SD	n/mean	%/SD	difference
Sex (male)		1,798	90.3%	14,503	90.8%	−0.018	563	97.2%	4,683	97.4%	−0.009
Age		52.0	7.3	50.6	7.6	0.202	51.0	7.2	48.8	7.4	0.303
BMI (kg/m^2^)		24.2	3.4	23.8	3.4	0.123	24.4	3.6	23.9	3.5	0.124
Abdominal circumference (cm)		86.0	8.9	84.8	8.7	0.132	87.0	9.2	85.4	9.1	0.171
Alcohol consumption per day*											
	20–40 g	136	6.8%	1,031	6.5%	0.015	–	–	–	–	
	40–60 g	1855	93.2%	14,937	93.5%	−0.015	–	–	–	–	
Over the threshold of referral for lifestyle-related diseases (threshold value)											
	Systolic blood pressure (140 mmHg)	1,117	56.1%	6,406	40.1%	0.324	298	51.5%	1,737	36.1%	0.313
	Diastolic blood pressure (90 mmHg)	1,179	59.2%	6,998	43.8%	0.312	313	54.1%	2,005	41.7%	0.249
	HbA1c (6.5%)	180	9.0%	853	5.3%	0.144	58	10.0%	248	5.2%	0.184
	Fasting blood glucose (126 mg/dl)	217	10.9%	1,045	6.5%	0.155	62	10.7%	307	6.4%	0.155
	Casual blood glucose (126 mg/dl)	18	0.9%	156	1.0%	−0.008	10	1.7%	37	0.8%	0.086
	GOT (51 U/L)	175	8.8%	1,344	8.4%	0.013	75	13.0%	590	12.3%	0.021
	GPT (51 U/L)	311	15.6%	2,341	14.7%	0.027	98	16.9%	855	17.8%	−0.023
	γGT (101 U/L)	791	39.7%	7,249	45.4%	−0.115	287	49.6%	2,610	54.3%	−0.094
	Triglycerides (300 mg/dl)	279	14.0%	1,723	10.8%	0.098	96	16.6%	676	14.1%	0.070
	HDL cholesterol (34 mg/dl)	33	1.7%	132	0.8%	0.075	6	1.0%	52	1.1%	−0.004
	LDL cholesterol (140 mg/dl)	585	29.4%	3,976	24.9%	0.101	148	25.6%	1,191	24.8%	0.018
	Uric acid (8.0 mg/dl)	159	8.0%	1,431	9.0%	−0.035	49	8.5%	548	11.4%	−0.098
Motivation: lifestyle change											
	No intention	496	24.9%	4,732	29.6%	−0.106	167	28.8%	1,558	32.4%	−0.077
	Intention within 6 months	656	33.0%	5,194	32.5%	0.009	180	31.1%	1,500	31.2%	−0.002
	Intention within 1 month	342	17.2%	2,508	15.7%	0.040	101	17.4%	763	15.9%	0.042
	Maintained for<6 months	176	8.8%	1,257	7.9%	0.035	51	8.8%	356	7.4%	0.052
	Maintained for over 6 months	279	14.0%	2,034	12.7%	0.037	71	12.3%	569	11.8%	0.013
Motivation: health guidance											
	Intention to receive health guidance	583	29.3%	4,365	27.3%	0.043	182	31.4%	1,371	28.5%	0.064

* By definition, all heavy drinkers had alcohol consumption of > 60 g/day.

HbA1c: hemoglobin A1c, GOT: glutamate oxaloacetate transaminase, GPT: glutamate pyruvate transaminase, γ-GT: gamma-glutamyl transpeptidase, HDL: high-density lipoprotein, LDL: low-density lipoprotein

### 3.2 Propensity score-matched participants and adjusted outcomes

Propensity score matching selected 1990 pairs of risky drinkers and 575 pairs of heavy drinkers (Table 2). The standardized differences between the groups were less than 0.1 for most of the variables. In both the risky drinkers and heavy drinkers, a majority of participants were male, the mean age was about 51 years, and the mean BMI was approximately 24. The major reasons for treatment recommendation were high blood pressure or high γ-GT. Detailed results of blood tests after propensity score matching are shown in Supplementary Table 3.

**Table 2:** Demographic characteristics and health conditions at the first health checkup after propensity score matching.

		Risky drinker (n = 3,980)					Heavy drinker (n = 1,150)				
		Treatment		No treatment			Treatment		No treatment		
		(n = 1,990)		(n = 1,990)		Standardized	(n = 575)		(n = 575)		Standardized
		n/mean	%/SD	n/mean	%/SD	difference	n/mean	%/SD	n/mean	%/SD	difference
Sex (male)		1,798	90.4%	1,780	89.5%	0.030	560	97.4%	560	97.4%	0.000
Age		52.0	7.2	52.0	7.7	0.008	51.0	7.2	51.0	7.4	0.007
BMI (kg/m^2^)		24.2	3.4	24.2	3.6	−0.020	24.4	3.6	24.3	3.4	0.027
Abdominal circumference (cm)		86.0	8.9	86.1	9.2	−0.013	86.9	9.2	86.8	9.0	0.017
Alcohol consumption per day*											
	20–40 g	135	6.8%	152	7.6%	−0.033	–	–	–	–	
	40–60 g	1,855	93.2%	1,838	92.4%	0.033	–	–	–	–	
Over the threshold of referral for lifestyle-related diseases (threshold value)											
	Systolic blood pressure (140 mmHg)	1,116	56.1%	1,114	56.0%	0.002	295	51.3%	314	54.6%	−0.066
	Diastolic blood pressure (90 mmHg)	1,178	59.2%	1,200	60.3%	−0.023	310	53.9%	314	54.6%	−0.014
	HbA1c (6.5%)	180	9.1%	197	9.9%	−0.029	56	9.7%	61	10.6%	−0.029
	Fasting blood glucose (126 mg/dl)	217	10.9%	224	11.3%	−0.011	61	10.6%	67	11.7%	−0.033
	Casual blood glucose (126 mg/dl)	18	0.9%	19	1.0%	−0.005	9	1.6%	9	1.6%	0.000
	GOT (51 U/L)	175	8.8%	190	9.6%	−0.026	74	12.9%	69	12.0%	0.026
	GPT (51 U/L)	311	15.6%	314	15.8%	−0.004	98	17.0%	93	16.2%	0.023
	γGT (101 U/L)	791	39.8%	814	40.9%	−0.024	286	49.7%	286	49.7%	0.000
	Triglycerides (300 mg/dl)	279	14.0%	269	13.5%	0.015	95	16.5%	97	16.9%	−0.009
	HDL cholesterol (34 mg/dl)	33	1.7%	30	1.5%	0.012	6	1.0%	10	1.7%	−0.059
	LDL cholesterol (140 mg/dl)	584	29.4%	589	29.6%	−0.006	147	25.6%	137	23.8%	0.040
	Uric acid (8.0 mg/dl)	159	8.0%	163	8.2%	−0.007	49	8.5%	48	8.3%	0.006
Motivation: lifestyle change											
	No intention	496	24.9%	468	23.5%	0.033	167	29.0%	184	32.0%	−0.064
	Intention within 6 months	655	32.9%	699	35.1%	−0.047	178	31.0%	173	30.1%	0.019
	Intention within 1 month	342	17.2%	346	17.4%	−0.005	101	17.6%	75	13.0%	0.126
	Maintained for<6 months	176	8.8%	156	7.8%	0.036	49	8.5%	52	9.0%	−0.018
	Maintained for over 6 months	279	14.0%	277	13.9%	0.003	71	12.3%	84	14.6%	−0.066
Motivation: health guidance											
	Intention to receive health guidance	582	29.3%	619	31.1%	−0.041	180	31.3%	179	31.1%	0.004

* By definition, all heavy drinkers had alcohol consumption of > 60 g/day.

BMI: body mass index, HbA1c: hemoglobin A1c, GOT: glutamate oxaloacetate transaminase, GPT: glutamate pyruvate transaminase, γ-GT: gamma-glutamyl transpeptidase, HDL: high-density lipoprotein, LDL: low-density lipoprotein

Table 3 shows the outcomes in the matched individuals. In the risky drinkers, individuals in the outpatient screening and treatment group were more likely to reduce the frequency of alcohol drinking than the screening alone group (11.7% vs. 8.7%, p = 0.002). However, there was no significant difference in the proportion of individuals who reduced consumption per day (26.1% vs. 25.7%, p = 0.772). The treatment group showed lower GOT (mean: 29.6 vs. 30.7, p = 0.034), lower GPT (mean: 30.8 vs. 32.2, p = 0.034), and lower γ-GT (mean: 97.6 vs. 102.8, p = 0.005). There was no significant difference in the proportion of individuals with liver dysfunction.

**Table 3:** Alcohol drinking patterns and liver function in subsequent annual health checkup after propensity score matching.

		Risky drinker (n = 3,980)					Heavy drinker (n = 1,150)				
		Treatment		No treatment		p	Treatment		No treatment		p
		(n = 1,990)		(n = 1,990)			(n = 575)		(n = 575)		
		n/mean	%/SD	n/mean	%/SD		n/mean	%/SD	n/mean	%/SD	
Alcohol: frequency											
	Improved	233	11.7%	173	8.7%	0.002^a^	51	8.9%	47	8.2%	0.673^a^
	No change/Worsened	1,757	88.3%	1,817	91.3%		524	91.1%	528	91.8%	
Alcohol: consumption/day											
	Improved	520	26.1%	512	25.7%	0.772^a^	231	40.2%	207	36.0%	0.145^a^
	No change/Worsened	1,470	73.9%	1,478	74.3%		344	59.8%	368	64.0%	
Liver function											
	Dysfunction	728	36.6%	768	38.6%	0.191^a^	253	44.0%	272	47.3%	0.261^a^
	GOT (U/L)	29.6	17.4	30.7	23.1	0.034^b^	33.1	26.3	34.6	22.4	0.146^b^
	GPT (U/L)	30.8	21.2	32.2	26.7	0.034^b^	32.9	23.3	34.6	26.4	0.134^b^
	γGT (U/L)	97.6	94.3	102.8	102.7	0.050^b^	119.8	115.2	130.9	127.8	0.061^b^

^a^ Chi-squared test. ^b^ Student's *t*-test

GOT: glutamate oxaloacetate transaminase, GPT: glutamate pyruvate transaminase, γ-GT: gamma-glutamyl transpeptidase

In the heavy drinkers, there were no significant differences in either the proportion of individuals who reduced the frequency of alcohol drinking (8.9% vs. 8.2%, p = 0.673) or the proportion of individuals who reduced consumption per day (40.2% vs. 36.0%, p = 0.145). There were no significant differences in either laboratory results or the proportion of individuals with liver dysfunction.

## 4 Discussion

We assessed changes in drinking patterns and evaluated liver functions in people who sought outpatient services after receiving a recommendation to receive outpatient treatment for lifestyle-related diseases following an alcohol consumption screening at a health checkup. The comparison group was comprised of people who were given a recommendation to receive outpatient treatment, but did not do so. In the risky drinkers, outpatient treatment was associated with a greater reduction in the frequency of alcohol drinking and lower GOT, GPT, and γ-GT at one year after a health checkup. In the heavy drinkers, however, there was no significant association between outpatient treatment and reduction in frequency or amount of drinking, and treatment was also not associated with improved liver function.

The majority of the participants were middle-aged males. Especially, over 95% of the heavy drinkers were male. A nationwide survey revealed that about 20% of middle-aged males were excessive drinkers with risks of lifestyle-related diseases, and this proportion was higher than those of other age groups (Ministry of Health, Labour and Welfare, 2020). Even though the participants of this study were the major targeted population of the intervention for excessive drinking and lifestyle-related diseases, only about 10% of the participants sought outpatient services after the recommendation to receive treatment. Approximately 30% of the participants answered at health checkups that they did not intend to change their lifestyle. Most appeared to be unmotivated to receive treatment.

In general treatment for lifestyle-related diseases, primary care physicians provide advice about prescription, diet, and exercise based on a treatment plan. If a patient has an alcohol problem, a physician helps the patient understand the risks or adverse effects of excessive drinking and advises on possible ways to reduce alcohol consumption. These interventions may guide the patient to improve drinking behavior. In addition, other interventions for lifestyle-related diseases, such as motivational advice to be physically more active, may also have indirect effects that improve drinking behavior. Among the risky drinkers evaluated in the present study, participants who received outpatient treatment were more likely to reduce the frequency of drinking compared to those without treatment. However, there was no significant reduction in the amount of drinking. In a meta-analysis assessing the effects of SBI on drinking patterns after one year, the mean reduction in the amount of drinking was 20 g/week (Kaner et al., 2018). Detection of a small change in alcohol consumption was difficult in the present study because we used a questionnaire that categorized alcohol consumption in increments of 20 g. In the heavy drinkers, outpatient treatment was not associated with a reduction in either the frequency or amount of drinking. The effectiveness of brief intervention for people with alcohol dependence or very heavy drinking is still unclear (Saitz, 2010). More intensive intervention may be needed to affect their drinking behavior.

In risky drinkers, outpatient treatment was associated with lower GOT, GPT, and γGT values in the next annual checkup, while there was no significant association between outpatient treatment and the proportion of individuals with liver dysfunction. Although outpatient treatment did not have a significant impact on altering the proportion of liver dysfunction, it may have reduced the amount of drinking and improved their markers for liver function. In heavy drinkers, there was no significant difference in liver dysfunction or values for liver conditions. For people with severe drinking problems, additional interventions should be considered because general management for lifestyle-related disease may not improve their liver function.

This study evaluated the effectiveness of screening of excessive alcohol consumption at health checkup settings and intervention during general treatment in Japan. Although referral to treatment in the usual SBI framework means referral to specialized treatment for alcohol dependence, general medical services may also be effective because hazardous and harmful drinkers are likely to have lifestyle-related diseases without alcohol dependence. Because an alcohol drinking habit is related to other risk factors of lifestyle-related diseases such as tobacco use, hypertension, and sleep problems (Prabhu et al., 2014, Simou et al., 2018, Hu et al., 2020, Chi et al., 2017, Timko et al., 2016), comprehensive screening and recommendation to receive general outpatient treatment may be useful for people with excessive alcohol consumption. This unique system in Japan has several benefits: provision of screening of alcohol drinking for all insured people, early detection of people at high risk of various lifestyle-related diseases associated with alcohol drinking, recommendation to receive accessible general medical services, and consecutive follow-up. Despite the recommendations for SBI, studies showed that very few patients in primary health care are screened and given advice regarding their excessive drinking (Bendtsen et al., 2015, Rehm et al., 2016). Routine annual health checkups could be useful for screening of alcohol drinking. To maximize the opportunity for intervention, it may also be useful to make recommendations to receive medical treatment based on the behavioral risk factor of high alcohol consumption. On the other hand, there may be people with alcohol dependence who cannot reduce alcohol consumption due to a brief intervention alone. Collaboration between general physicians and psychiatrists providing specialized treatment for alcohol dependence is recommended for these severe patients (Hargraves et al., 2017, Muto and Yzuriha, 2015).

This study has some possible limitations. First, the participants who received treatment for lifestyle-related diseases after the health checkup screening might also be highly motivated to improve drinking behavior. Although we used their intention to change lifestyles for adjustment, the variable was for intention to change lifestyles in general and not specifically alcohol drinking behavior. There could also be residual confounding. Second, actual treatment for lifestyle-related diseases was provided by different clinics or physicians. Intervention at outpatient treatment in this study is different from a typical brief intervention because treatment intensity for alcohol-related problems might depend on clinics or physicians. Third, some participants may have received health guidance from healthcare professionals during health checkups. However, we could not analyze data regarding health guidance. Fourth, the frequency and amount of drinking were self-reported and could be underreported. Additionally, drinking patterns were assessed by categories of frequency and amount. People with different patterns of consumption could be included in the same category, and people with episodic binge drinking could not be identified. Lastly, the study population consisted of predominantly male employees. Therefore, generalizability to females may be limited.

## 5 Conclusion

This study suggested that general outpatient treatment for lifestyle-related diseases following screening for excessive alcohol consumption at health checkups may reduce the frequency of alcohol drinking and improve liver function among risky drinkers after one year. Further study is needed to evaluate the effectiveness of screening and general treatment for heavy drinkers.

## Author contributions

AT, HaY, SO, and HiY devised the study protocol. AT, HaY, SO, HM and HiY contributed to data collection and analysis and drafted the manuscript. All authors have contributed to interpretation and critically reviewed the manuscript. All authors approved the final version of the manuscript.

## Declaration of Competing Interest

The authors declare that they have no known competing financial interests or personal relationships that could have appeared to influence the work reported in this paper.
